# Apical ballooning syndrome after attempted suicidal hanging

**DOI:** 10.4103/0972-5229.78225

**Published:** 2011

**Authors:** Jose Chacko, Gagan Brar, Ashok Elangovan, Ramanathan Moorthy

**Affiliations:** Multidisciplinary Intensive Care Unit, Manipal Hospital, Bangalore, India

**Keywords:** Apical ballooing, left ventricular dysfunction, suicidal hanging

## Abstract

We report a case of “Apical Ballooning Syndrome” following attempted suicidal hanging. Our patient developed retrosternal chest pain and ischemic changes on electrocardiography (ECG), a day after the suicidal attempt. She underwent an angiogram considering the possibility of acute coronary syndrome. However, her coronary arteries were normal; the left ventricle showed the typical ballooning pattern characterized by hypokinesia of the distal septum and apex. On follow-up a week later, she remained asymptomatic; her ECG changes had reversed and the left ventricular contractility was normal on echocardiography.

## Introduction

The “Apical Ballooning Syndrome” (ABS), characterized by transient, reversible hypokinesia of the distal interventricular septum and the apex of the left ventricle (LV), with normal coronary arteries was first reported in the Japanese population.[1] Subsequently, there have been reports of this syndrome in situations associated with extreme physical or emotional stress.[2] We report this syndrome in a 45-year-old woman following attempted suicidal hanging.

## Case Report

**Figure 1:**
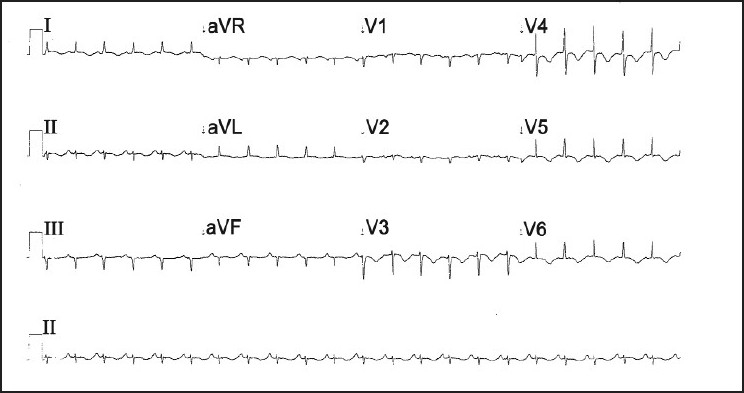
ECG showing T wave inversion in I, AVL and V2–V6

**Figure 2:**
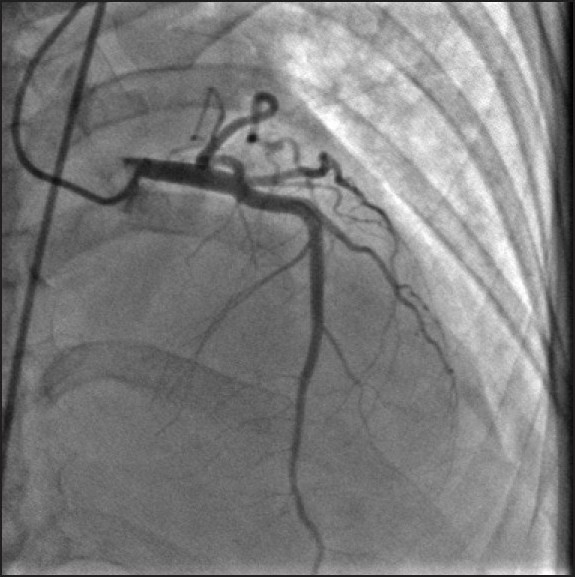
Coronary angiogram showing normal coronary arteries

**Figure 3:**
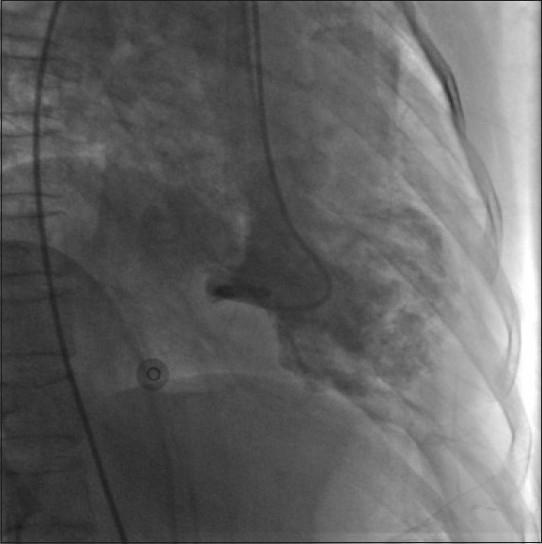
Ventriculogram showing ballooned out apex and hypercontractile basal segment

A 45-year-old woman presented to our accident and emergency department after attempted suicidal hanging. On initial evaluation, she was in respiratory distress with heart rate 110/min and blood pressure (BP) 100/70 mm Hg. A ligature mark was clearly visible around the neck. She had no eye opening and did not respond to verbal commands. Her pupils were 3 mm each and reacting to light and she was decerebrating spontaneously. Her arterial blood gas analysis was within normal limits. She was intubated, ventilated and maintained on an infusion of fentanyl and propofol for the next 24 hours. Apart from sinus tachycardia, she remained hemodynamically stable and maintained normal blood gases. The following morning, she was fully awake and appropriate after stopping all sedation and was extubated uneventfully. However, she remained tachycardic, and a few hours later, started complaining of retrosternal chest pain. Her heart rate was 130/min with stable BP. An S3 gallop could be heard over the apex. A 12-lead ECG revealed T wave inversion in leads V_2_-V_6_, Lead I and AVL [Figure 1]. An echocardiogram showed segmental hypokinesia of the distal septum and apex with “ballooning out” of the left ventricular apex. There was no evidence of dynamic left ventricular outlet obstruction or mitral regurgitation. The right ventricle was normal in size and function. Her troponin T was negative and the creatinine kinase (CK) level was normal. Suspecting acute coronary syndrome, she underwent a coronary angiogram. Her coronary arteries did not show any evidence of occlusion [Figure 2]; ventriculography showed a hypokinetic, ballooned out left ventricular apex with a hypercontractile basal segment [Figure 3]. She was put on metoprolol, with which her heart rate settled to 100/min. Her chest pain eased off in the next few hours and she was discharged home after 5 days in hospital. On follow-up a week later, her ECG had returned back to normal. The echocardiogram revealed a normally contracting apex and distal septum and the overall left ventricular function had returned back to normal, with an ejection fraction of 60%.

## Discussion

Transient LV dysfunction characterized by segmental hypokinesia of the apex and distal septum, with normal coronary arteries was first described in the Japanese population by Sato *et al*. who called it the Tako Tsubo syndrome for the typical appearance on left ventriculogram resembling an octopus trap with a balloon like bottom and a narrow neck.[1] ABS is being increasingly recognized under situations of extreme emotional and physical stress and in association with conditions like subarachnoid hemorrhage.[2] There appears to be a marked female predilection with over 88% of subjects being women in a systematic review.[3]

The etiology of stress-induced cardiomyopathy is unclear. Extremely high catecholamine levels, though not consistently found, seem to be a triggering factor.[4] Excessive catecholamine stimulation may result in direct cardiac myocyte toxicity and also lead to microvascular endothelial dysfunction or spasm.[5] The apex may be more vulnerable to excessive sympathetic stimulation due to lack of the typical three-layered architecture, reduced elastic properties and a limited coronary circulation.[6] Myocardial perfusion studies using single photon emission computed tomography (SPECT) and technetium-99 tetrofosmin tomographic myocardial imaging indicate reversible myocardial ischemia in ABS, in the absence of coronary artery occlusion.[7]

We came across four other reports of transient LV dysfunction associated with attempted suicidal hanging or accidental strangulation. In three of these cases, the echocardiogram revealed global hypokinesia rather than the apical and distal septal involvement that is characteristic of ABS.[8,9,10] Sivanandan *et al*. reported a case of apical akinesia and globally impaired left ventricular function in an 8-year-old boy following accidental strangulation.[11] To our knowledge, this is the only reported case, apart from ours, that presented with the typical echocardiographic findings of ABS. It is important to distinguish between the truly focal hypokinesia seen in ABS from the global impairment of left ventricular function that occurs with extreme emotional or physical stress although the underlying pathophysiological mechanism may be the same.

Our patient developed tachycardia and retrosternal chest pain with ischemic changes on the ECG, following attempted suicidal hanging. We initially considered an acute coronary syndrome; however, her coronary arteries were normal on angiogram. Revascularization can occur after spontaneous lysis of a left anterior descending artery thrombus with similar ECG and echocardiographic features; however, we considered this less likely as the apical hypokinesia did not conform to the typical anatomical distribution. Indeed, wall motion abnormalities that occur beyond the distribution of any single coronary artery is a feature of ABS.[12] The CK and troponin T levels were not elevated in our case; disproportionately low levels of biomarkers for the extent of left ventricular involvement is a feature of ABS.[13] Transient epicardial coronary artery spasm may cause ECG changes and wall motion abnormalities on the echocardiogram; however, the dyskinetic pattern would be confined to the known anatomical territory of a coronary artery.[3] ECG changes of acute ischemia, modest elevations of cardiac enzymes and reversible segmental wall motion abnormalities are seen in acute lymphocytic myocarditis; however, the typical ballooning out of the left ventricular apex was not evident in biopsy proven cases.[14] Besides, no histopathological evidence of myocarditis was demonstrated in cases of ABS wherein an endomyocardial biopsy was done.[15]

Pulmonary edema is well known to occur after attempted hanging.[16] Many of these reports attribute the sudden onset pulmonary edema to airway obstruction. However, recent analysis of video recordings of hanging episodes clearly demonstrate continued unobstructed respiratory efforts.[17] It is possible that at least some of the reports of presumed post-obstructive pulmonary edema may in fact be due to transient global or segmental left ventricular hypokinesia.

The optimal treatment of ABS is unclear. It would seem intuitive to administer beta blockers in a situation characterized by intense sympathetic activity. A reduction in the gradient between the apex of the left ventricle and outflow has been observed with intravenous propranalol in ABS.[18] Our patient went on to make an uneventful recovery and had complete reversal of her ECG and echocardiographic findings on follow-up a week after the event. Favorable outcomes have been reported overall with supportive treatment in ABS with an in hospital mortality of 0–8%.[19]
